# Brazil is getting older: some lessons from the Bambuí Health and Aging Study

**DOI:** 10.1590/S1516-31802004000300001

**Published:** 2004-05-06

**Authors:** Paulo Andrade Lotufo

**Figure 1:**
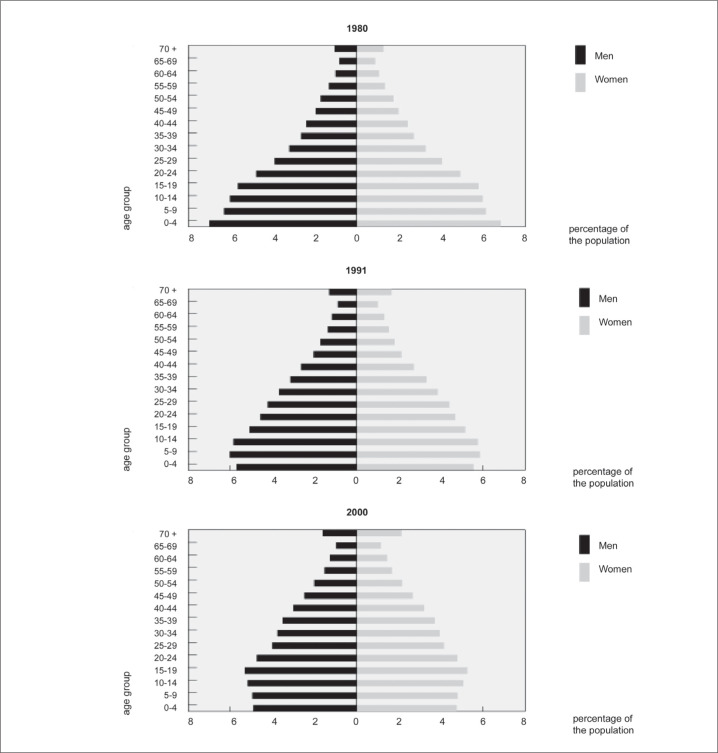
Proportional distribution (%) of the Brazilian population by sex and age, in 1980, 1991 and 2000, according to the Brazilian Institute of Geography and Statistics (Instituto Brasileiro de Geografia e Estatística, IBGE).

The difference in Brazil's age structure between 1980 and 2000 is very impressive, as shown in Figure 1. To demonstrate the process of aging that has taken place in this country, the proportion of the population aged over 60 years has increased from 6.1% to 8.6%. In 1980, no Brazilian State had more than 10% of its population in this age stratum. In 2000, the States of Rio de Janeiro (10.7%) and Rio Grande do Sul (10.5%) reached this level. The cities of Rio de Janeiro (12.7%) and Porto Alegre (11.8%) are the state capitals with the greatest numbers of elderly people. The proportion of the Brazilian population aged over 80 years in 2000 (1.1%) was twice what it was in 1980 (0.5%).

Contrary to common sense, the population aging process is due to the decline in fertility rather than lower mortality rates. To back up this statement, a comparison between the demographic dynamics of the United Kingdom (UK) and Brazil is very interesting. The total fertility rate (TFR) started to decline in the UK from 1870 onwards, from 5.3 births/woman aged 15-49 years, to reach a TFR of 2.2 by 1970. This was a decrease of 58% over one century. In Brazil, the TFR was 5.8 in 1970 (not 1870 as in the UK), and this had declined to 2.3 by 2000, i.e. a 60% reduction over only 30 years.^1^

Obviously, this demographic change is having an important impact on all aspects of health statistics and medical care. Recently, several papers have been published from a cohort of 1,742 citizens of Bambuí, a small town in Minas Gerais. All of these people are over 60 years old and they have been followed up yearly since 1996. The conclusions from these studies have been useful in understanding the burden of increasingly elderly people in Brazil.^2^ Lower family income was independently associated with less consumption of fresh fruits or vegetables and less frequent exercise during leisure time, inability to perform routine activities because of a health problem, higher quantities of non-prescription medications and higher numbers of hospitalizations.^3^ The prevalence of obesity was 12.5% and was positively associated with female gender, family income, hypertension and diabetes and inversely related to physical activity. Underweight affected 14.8% of the participants, increased with age and was higher among men and low-income families. Both conditions were associated with adverse effects on health.^4^

Although the Bambuí area was, in the past, an endemic area for Chagas’ disease, the burden of classic cardiovascular diseases (i.e. heart diseases) described in this town, originating from high blood pressure and atherosclerosis, is higher than would have been expected due to Chagas’ disease. Almost half of the population between 60 and 75 years old will have a 20% chance of developing coronary heart disease over the next ten years, on the basis of the Framingham Heart Study scoring for predicting heart disease.^5^ Another important matter is the increase risk of end-stage renal failure, considering the levels of serum creatinine directly associated with age.^6^

The reasons for the excessive hospitalization among the elderly were determined in the Bambuí Health and Aging Study. The strongest associations for multiple hospital admissions were with living alone, financial constraints on purchasing medications and various indicators of need (worse self-perceived health, more visits to physicians, greater use of prescription medications and a history of coronary heart disease).^7^ More specifically, lower body-mass index and higher levels of serum cholesterol were associated with two or more hospital admissions.^8^

The Bambuí Health and Aging Study and other population-based studies in our country will certainly bring us new knowledge regarding the challenges faced in managing public health and medical care in Brazil.
